# Imaging of COVID-19-associated rhino-orbital-cerebral mucormycosis: imaging analysis of 120 patients

**DOI:** 10.1186/s43163-022-00342-7

**Published:** 2022-12-03

**Authors:** Alka Agrawal, Yogita Dixit, Vivek Yonati, Prakhar Nigam, Pramita Kheti

**Affiliations:** 1 Department of Radiodiagnosis, M.G.M. Medical College and M.Y. Hospital, CRP line, Indore, Madhya Pradesh 452001 India; 2 Department of Otorhinolaryngology, M.G.M. Medical College and M.Y. Hospital, Indore, India

**Keywords:** Mucormycosis, Magnetic resonance imaging, COVID-19, Invasive fungal sinusitis

## Abstract

**Background:**

With the ongoing pandemic of COVID-19, there has been a rapid upsurge in cases of rhino-orbital-cerebral mucormycosis (ROCM). It is an opportunistic fungal infection associated with high morbidity and mortality. Rapid and appropriate application of clinical and radiological methods is crucial for early diagnosis, to limit the associated morbidity and improve post-treatment outcomes. In our study, we analyzed imaging features, common sites, and the extent of infection in patients suffering from ROCM.

**Results:**

The majority of the patients were either diabetics or developed uncontrolled blood glucose levels during COVID-19 infection. 79.17% of patients had a history of treatment with steroid therapy. Headache and facial pain were the most common clinical features seen in 76.67% and 60% of patients, respectively. Maxillary and ethmoid sinuses were commonly involved. The most common extra-sinus site of involvement was periantral fat and orbit, seen in 91 (75.83%) and 84 (70%) patients, respectively. Bone erosion or marrow edema was seen in 72 (60%) patients. Intracranial extension in the form of meningitis, cavernous sinus thrombophlebitis/thrombosis, and brain abscess were seen in 20%, 10%, and 3.3% of patients, respectively. MRI-based staging showed that 24.7% of patients had stage I, 5.83% had stage II, 50% had stage III, and 20% had stage IV disease.

**Conclusion:**

The spread of COVID-19-associated rhinomucormycosis to extra-sinus sites is common, which can be detected adequately on MRI. The radiological signs of invasion and devitalization of tissues are crucial for the early diagnosis of ROCM.

## Background

The coronavirus disease 2019 (COVID-19) infection caused by the novel severe acute respiratory syndrome coronavirus 2 (SARS-CoV-2) may be associated with a wide range of disease patterns, ranging from mild to life-threatening pneumonia [1]. A wide range of bacterial and fungal co-infections may exist, among which fungal is ten times more common. Mucormycosis is one of the most lethal types of zygomycosis that commonly occurs in post-COVID-19 patients. In the pre-COVID era, the incidence of mucormycosis was more common in India as compared to the western world [2]. With the second wave of COVID-19 in India, SARS-CoV-2 has further exaggerated the disease epidemiology.

India has a high prevalence rate of type 2 diabetes mellitus (8.9% of adults, 77 million patients), which is a well-known risk factor [3]. A complex interplay between COVID-19-induced immune alterations causing reduced CD4+ and CD8+ T-cell counts, impaired glycemic controlled diabetic COVID-19 patients treated with steroids, rampant use of immunosuppressive therapy (i.e., corticosteroids), broad-spectrum antibiotics, prolonged hospital stays, oxygen therapy, and ventilatory support has been proposed to be responsible for this superinfection [4, 5]

In most cases, infection occurs through inhalation of spores that invade the mucosa of the nasal cavity and sinuses, resulting in rhinosinusitis, followed by the involvement of extra-sinus soft tissue and the orbit. With the intracranial extension of disease, mortality is greater than 80% [6]. Fulminant progression is common and can result in death in less than a week after the initial presentation.

The most important factors that determine prognosis are the timing and efficacy of treatment. The role of imaging in ROCM lies in the early identification of infection when it is confined to paranasal sinuses, early detection of extra-sinus spread, and identifying the extent of infection to determine the limit of surgical debridement. Furthermore, for patients with clinical suspicion and imaging evidence of ROCM, empirical antifungal therapy can be initiated even before microbiology or histopathology confirms the diagnosis.

In order to radiologically diagnose a patient with this invasive fungal infection, one must be aware of the common and characteristic appearance of the lesion. Our study aims to identify and describe the lesions seen in rhino-orbital-cerebral mucormycosis and the site and pattern of the spread of infection.

## Methods

After approval from the institutional scientific and ethics review committee, we retrospectively analyzed imaging data of 120 confirmed cases of rhino-orbital-cerebral mucormycosis, who presented between May 2021 and September 2021. All included patients had microbiological or histopathological evidence of invasive sinonasal fungal infection by species belonging to order Mucorales. All patients had a history of COVID-19 illness confirmed by either reverse transcriptase-polymerase chain reaction of nasopharyngeal swab or computed tomography of the chest. Exclusion criteria were patients with a glomerular filtration rate (GFR) of <40, patients with claustrophobia, and MRI-incompatible implants.

A detailed history of the patient and physical examination findings were obtained by studying patients’ medical records. All patients underwent MRI on a 3T, 97 channel system. Following sequences had been performed in the MR evaluation of all the patients—unenhanced axial and coronal T1- and T2-weighted images, axial fluid-attenuated inverse recovery sequence (FLAIR), and fat-saturated T1- and T2-weighted images as well as sagittal T2-weighted images. The study also included contrast-enhanced and diffusion-weighted imaging (DWI; b values of 50 and 1000). CT correlation was done for those patients who underwent CT scan using a 128-section CT multisection scanner with image reconstruction in soft-tissue and bone windows. Results were analyzed at the workstation. The radiological images were studied to look for common sites and the signal intensities of the lesions along with the changes in surrounding tissues. Based on the radiological extent, the severity of the disease was graded into four stages [7]. Data were tabulated in a Microsoft Excel sheet, for the analysis of data. Further depiction of data was done in the form of various tables and charts. IBM Statistical Package for the Social Sciences (SPSS) software for Windows, Version 26.0. Armonk, NY: IBM Corp. SPSS was used to analyze the data. The mean and standard deviation of the quantitative variables were calculated.

## Results

Out of 120 patients, two-thirds were males (66.67%). The most common age group affected was in the range of 41–50 years. Eighty patients were known diabetics while 35 developed uncontrolled blood glucose levels during COVID-19 illness. The remaining five patients had a history of hematological malignancies. Ninety-five patients (79.17%) received steroids and 78 patients (65%) received oxygen support for treatment of COVID-19. The onset of symptoms of mucormycosis after contracting COVID-19 was in the range of 2 weeks to 18 weeks.

Headache, facial/periorbital pain, and swelling were the most common presenting complaints seen in 76.67%, 60%, and 58.33% of patients, respectively. Maxillary and ethmoid sinuses were affected in 95.83% and 89.17% of patients, respectively. Sphenoid and frontal sinuses were infected in 75% and 45.83% of patients, respectively.

The signal intensities on T1- and T2-weighted MR imaging had various appearances. The majority of the lesions showed heterogeneous or hypointense signal on T2 sequence with peripheral enhancing and central non-enhancing areas on the administration of contrast (Table 1)

**Table 1:** MR features of sino-nasal lesions

T1-weighted imaging		
Isointense/hypointense	120	100%
T2-weighted imaging		
Isointense/hypointense	39	32.50%
Heterogenous	58	48.33%
Hyperintense	23	19.67%
Enhancement pattern		
Intense homogenous	5	4.17%
Heterogenous	20	16.67%
Central non-enhancement with peripheral enhancement	88	73.33%
Complete non-enhancement	7	5.83%

**Fig. 1:**
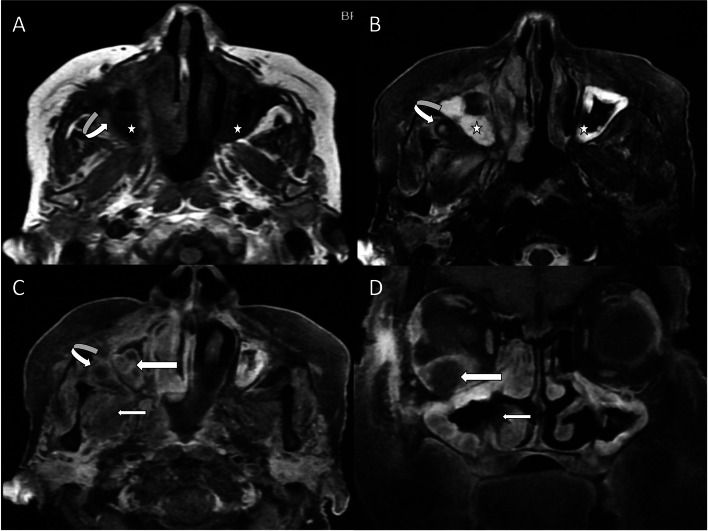
Sinu-nasal mucormycosis with extension into retroantral fat, pterygoid muscle, and orbit. Axial T1-weighted image (**A**) shows hypointense mucosal thickening of the walls of bilateral maxillary sinuses (white stars) with the erosion of posterior wall of right maxillary sinus (curved arrow). Axial T2-weighted fat-saturated image (**B**) shows a well-defined hypointense lesion in right retroantral region (curved arrow) with central hyperintensity. Mucosal thickening appears hyperintense (white stars). Axial contrast-enhanced fat-saturated T1-weighted image (**C**) shows peripheral enhancement of the sinus mucosa (straight long arrow) and right retroantral lesion (curved arrow). Note the involvement of the right pterygoid muscle (straight short arrow) which appears bulky and shows heterogeneous enhancement. Coronal contrast-enhanced fat-saturated T1-weighted image (**D**) shows a peripheral enhancing lesion in the right orbit with central non-enhancing area (straight long arrow) suggesting intraorbital abscess formation. Part of the right inferior turbinate (straight short arrow) shows no enhancement (the “Black turbinate sign”)

The most common extra-sinus site of involvement was periantral fat (Fig. 1), followed by orbit in the form of extraconal or intraconal compartment fat stranding with or without orbital apex involvement (Table 2). Bone involvement was seen in 72 patients (60%), most commonly in the maxillary sinus walls and lamina papyracea.

**Table 2:** Various sites of extra-sinus involvement

Site involved	Number (percentage)
No extra-sinus extension	29
Maxillo-facial soft tissue extension	91 (75.83%)
Periantral fat (preantral and retromaxillary)	91 (75.83%)
Masticator space	60 (50%)
Infratemporal fossa	47 (39.17%)
Pterygopalatine fossa	36 (30%)
Buccal space	8 (6.67%)
Maxillo-facial bone involvement	72 (60%),
Orbital involvement	84 (70%)
Extraconal fat	82 (68.83%)
Extraoccular muscles	32 (26.67%)
Intraconal fat	31 (25.83%)
Orbital apex	20 (16.67%)
Intracranial involvement	
Meninges	24 (20%)
Cavernous sinus	12 (10%)
Internal carotid artery	1 (0.83%)
Cerebritis	3 (2.5%)
Cerebral abscess	4 (3.33%)
Cerebral infarction	6 (5%)

Out of 120 patients, CT images were available for 38 patients. The involved sinuses showed nodular thickening of mucosal walls with the absence of air-fluid levels. Bony erosions were seen in 28 patients, while premaxillary/retroantral fat stranding was present in 35 patients.

**Fig. 2:**
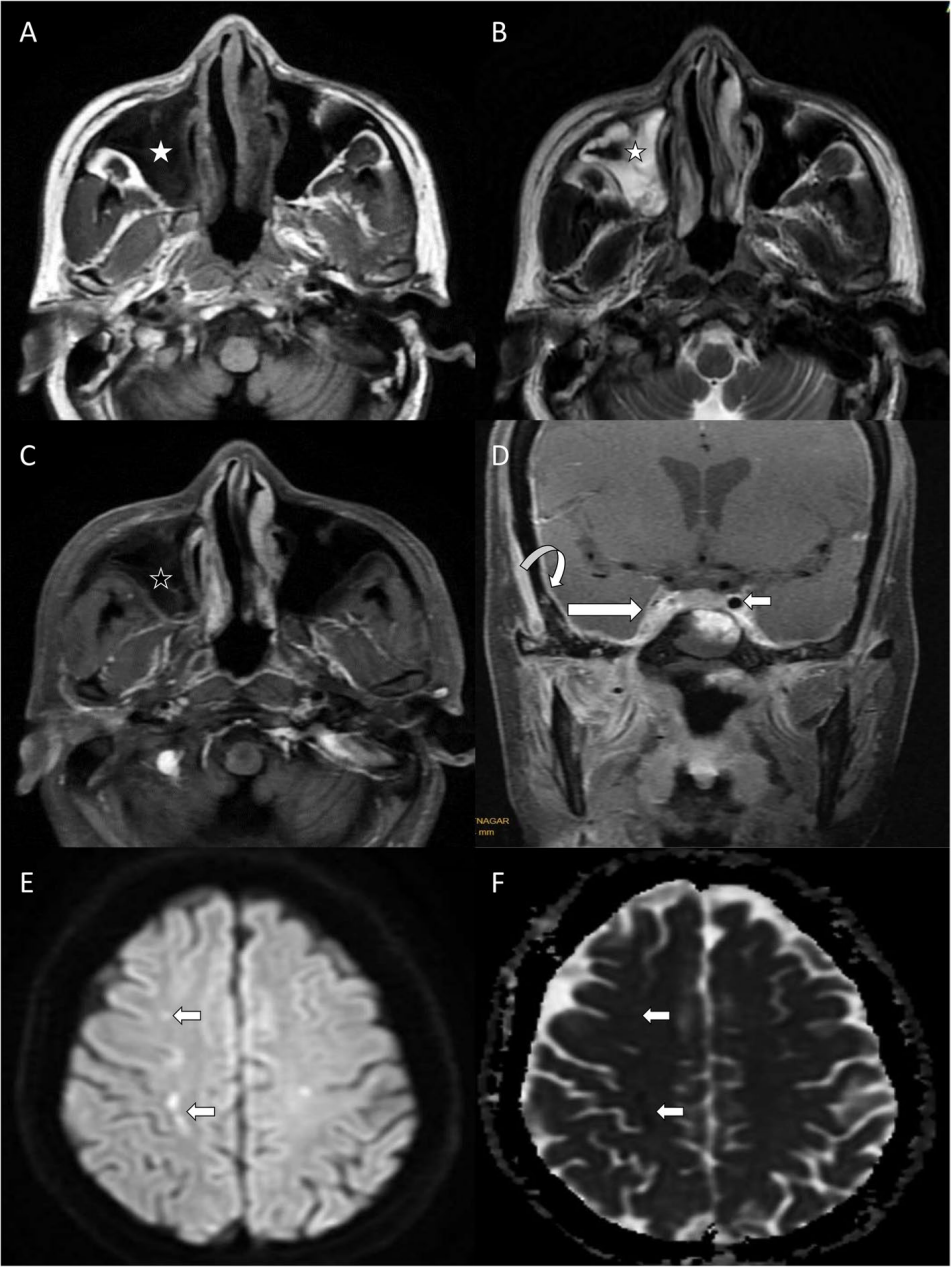
Extension of sinonasal disease into the cavernous sinus. Axial T1- and T2-weighted images (**A** and **B**) show thickening of the right maxillary sinus wall (white stars) which shows no enhancement (black star) on axial contrast-enhanced fat-saturated T1-weighted image (**C**). Coronal contrast-enhanced fat-saturated T1-weighted image (**D**) shows non-enhancing areas within the right cavernous sinus with convex outward margin (straight long arrow), suggestive of cavernous sinus thrombosis. The flow void of the cavernous part of the right internal carotid artery is not seen (straight white arrow shows normal flow void of the internal carotid artery on the left side). The meninges appear thickened and show enhancement (curved arrow) indicating meningitis. Axial diffusion-weighted (**E**) and apparent diffusion coefficient (**F**) images show foci of restricted diffusion (straight short arrows) in the right centrum semiovale in the watershed territory, suggesting cerebral infarcts

**Fig 3:**
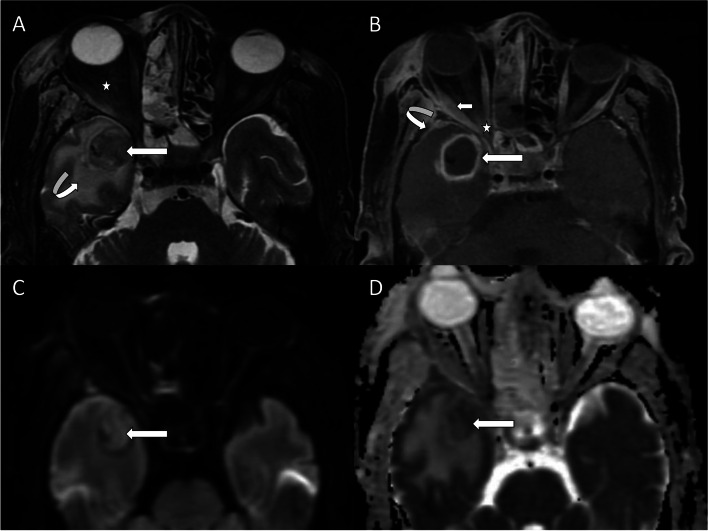
Orbital and cerebral involvement in ROCM. Axial fat-saturated T2-weighted image (**A**) shows a well-defined hypointense lesion (straight long arrow) in the right temporal lobe with surrounding edema (curved arrow). Right intraconal fat (white star) shows stranding with associated proptosis of the right eyeball. Axial contrast-enhanced fat-saturated T1-weighted image (**B**) shows peripheral enhancement of the temporal lobe lesion (straight long arrow) along with the enhancement of meninges (curved arrow). The right lateral rectus muscle appears bulky with heterogeneous enhancement (straight short arrow). Also note enhancing soft tissue in the right orbital apex (white star). The temporal lobe lesion (straight white arrows) in axial diffusion-weighted (**C**) and apparent diffusion coefficient (**D**) images shows central diffusion restriction, suggestive of an intracerebral abscess

On contrast-enhanced MR images, meningitis in the form of thickening and enhancement of dura was seen in 24 (20%) patients. Cavernous sinuses were involved in 12 patients (Fig. 2). One of these patients had a thrombus in the cavernous part of the internal carotid artery with an associated infarct in the middle cerebral artery territory. Cerebral infarcts were seen in five other patients. However, the sites of vessel occlusion could not be identified. None of them underwent angiographic studies. Intracerebral abscesses were noted in 4 patients in the anteromedial part of the temporal lobe (Fig. 3).

On staging the patients on the basis of MRI findings, 29 (24.17%) had disease confined to the nasal mucosa and/or paranasal sinuses. The majority of the patients with maxilla-facial soft tissue extension (84 out of 91) also had orbital and/or intracranial lesions at the time of imaging (Table 3).

**Table 3:** MRI staging of patients with COVID-19-associated mucormycosis

Staging	Site of involvement	Number of patients (percentage)
Stage I	Involvement of the nose and paranasal sinuses with no extra-sinus extension	29 (24.17%)
Stage II	Extra-sinus maxillo-facial extension	7 (5.83%)
Stage III	Extra-sinus maxillofacial and orbital extension with no intracranial involvement	60 (50.00%)
Stage IV	Intracranial extension	24 (20.00%)

## Discussion

Rhino-orbital-cerebral mucormycosis is caused by the infection of fungi belonging to the order Mucorales, which are usually present in soil and vegetative matter. Inhalation of spores of these fungi followed by colonization of nasal mucosa is the primary route of infection. The aggressive extra-sinus spread through direct extension, bony destruction, or perineural/perivascular routes leads to high mortality in infected patients.

Immunocompromised state, hematological malignancies, uncontrolled diabetes mellitus, and steroid therapy predispose individuals for mucormycosis [8]. In our study, the majority of the patients were either diabetic or developed uncontrolled blood glucose levels during COVID-19 infection. COVID-19 infection itself causes impairment of cell-mediated immunity through immunomodulation which makes patients susceptible to fungal co-infections. History of steroid administration, oxygen therapy, and prolonged hospital stay was common in our studied population. Thus, both COVID-19 illness and its treatment make patients vulnerable to opportunistic fungal infections.

Patients with ROCM may present with nonspecific symptoms of sinusitis such as headache, facial pain, fever, nasal discharge, or congestion. Orbital symptoms such as swelling, proptosis, visual disturbances, and ophthalmoplegia or neurological symptoms such as confusion and altered sensorium should raise the suspicion of invasive fungal sinusitis, especially in the presence of predisposing factors. In the era of COVID-19 infection, all patients with sinusitis should be properly evaluated clinically, radiologically, and if required, histopathologically to rule out ROCM.

On imaging, there is variation in MR signals on T1- and T2-weighted imaging. However, the presence of low signals on T2-weighted imaging characteristically represents iron- and manganese-containing fungal elements. In contrast studies, ROCM shows multiple enhancement patterns. In the early stages, intense homogenous enhancement of mucosa is seen, which is similar to bacterial sinusitis. Presence of heterogenous enhancement or non-enhancing areas within the lesion points towards invasive fungal etiology. The disease is known to cause devitalization of sinonasal mucosa by vascular invasion and infarction which corresponds to non-enhancing tissues on contrast administration. The “black turbinate sign,” which is an early sign of ROCM, simply represents the non-enhancing turbinate on contrast MR imaging [9, 10]. The non-enhancement should persist on delayed images unlike the benign black turbinate, which shows a gradual increase in signal.

Periantral fat invasion which may appear as stranding of premaxillary/retroantral fat, increased signals on fat-saturated T2-weighted imaging, or enhancement on contrast sequences is also an early feature of ROCM. Fat stranding is also well visualized on CT images. In our study, 75.83% of the patients showed involvement of periantral fat. A subset of patients with periantral extension had no bony erosion suggesting spread through perivascular routes. Bony erosion or marrow edema was present in 60% of patients. Erosions were commonly present in walls of maxillary antrum and orbital walls, allowing spread in retromaxillary soft tissue and orbit, respectively. The erosion or rarefaction of bony walls was better visualized on CT images.

Extension of infection in masticator space and infratemporal fossa was common in our study. Muscles of mastication appeared bulky with heterogeneous enhancement in involved cases. Pterygopalatine fossa was involved in about one-third of the studied cases. It is a neurovascular crossroad that serves as a route to infection into the middle cranial fossa.

Extension of infection into the orbit can be seen in the form of stranding of intraconal or extraconal fat, the presence of abnormal T2 hypointense soft tissue with variable enhancement, and/or bulky heterogeneous or non-enhancing intraocular muscles. There may not be associated erosion of orbital walls as infection can spread via nasolacrimal duct and vascular channels. Direct spread of infection through ethmoid and maxillary sinuses causes lateral displacement of the medial rectus and upward displacement of the inferior rectus, respectively. In our study, 24 patients out of 84 with orbital involvement had no specific ocular complaints. Therefore, it is advisable to include imaging of the orbit along with sinuses, in all suspected cases of ROCM.

The periantral soft tissue and orbits were the most common sites of disease extension in our study. The study conducted by Therakathu et al. [11] also showed orbits as the most common site of extra-sinus spread seen in 76% of patients, followed by facial soft tissue (57%). Metwally et al. [7] also found periantral soft tissue involvement in 74.6% of patients. However, pterygopalatine fossa invasion was more common in their study (77.8%). Yadav et al. [12] in their study on 50 patients with mucormycosis found periantral soft tissue and orbital involvement in 74% and 76% of patients, respectively. The close proximity to the commonly infected maxillary sinus, direct invasion through bony erosion, and tendency to spread along perineural/perivascular routes possibly explain the frequent involvement of periantral fat. The orbits are separated from sinonasal mucosa through thin lamina papyracea and communicate to the nasal cavity via the nasolacrimal duct. Thus, orbits are also anatomically prone to invasive fungal infection.

The presence of enhancing soft tissue at the orbital apex may cause involvement of optic nerve with a risk of extension of infection into the cavernous sinus. Optic nerve involvement leads to irreversible vision loss and is an ominous sign. The spread of infection in the cavernous sinus may lead to thrombophlebitis or thrombosis of the sinus. Various divisions of trigeminal nerves and other cranial nerves traversing the cavernous sinus may get involved leading to extension into the posterior cranial fossa [12]. The walls of the cavernous portion of the internal carotid artery show thickening and enhancement if involved, with or without the presence of a thrombus in the vessel lumen.

In our study, meninges were the most commonly involved intracranial structure, which appeared thickened with post-contrast enhancement. The involvement of meninges is the earliest sign of the intracranial spread of infection. Although intracranial spread through perineural or perivascular routes may not always show meningeal enhancement. Invasion of the brain can lead to the formation of fungal abscess which is common in the temporal lobe. It has a characteristic low signal on both T1- and T2-weighted sequences. The walls of these lesions show enhancement on contrast images and restricted diffusion on diffusion-weighted imaging (DWI). DWI is also useful in the identification of cerebral infarcts which occur due to the angioinvasion property of mucor. The pathology of mucormycosis is characterized by the proliferation of angioinvasion hyphae with the elastic lamina of large or medium-sized vessels [13], which necessitates the use of angiographic studies in suspected cases for meticulous assessment of vasculature.

On classifying the cases on the basis of MRI findings in our study, half of the patients were found in orbital stage (stage III disease), while only 7 patients had stage II disease. The majority of the patients with orbital invasion also had soft tissue lesions in periantral region and various neck spaces, suggesting there occurs rapid invasion of orbits post-maxillofacial soft tissue infection. Simultaneous invasion of orbits and periantral region is another possibility. If the latter is considered, then stage II and stage III disease can be clubbed together in a three-stage classification as proposed by Therakathu et al. and Yadav et al. However, despite a multitude of recent literature on rhino-orbital-cerebral mucormycosis, no consensus on imaging-based classification has been reached.

Nevertheless, imaging is indispensable for management in patients with rhino-orbital-cerebral mucormycosis (ROCM). Imaging is requisitioned not only for early diagnosis but for a variety of reasons that include pre-surgical mapping to detect early orbital involvement and demonstrate possible extension to the skull base, anterior, and middle cranial fossa, which may be clinically silent, and for guided biopsy to ensure maximum diagnostic yield.

Rapid and progressive intracranial spread of the mucor infection occurs either by direct extension across the neural foramina, cribriform plate/ethmoid, walls of the frontal and sphenoid sinuses, or angioinvasion of the walls of the arteries and veins, causing vascular thrombosis, occlusion, and infarction [6, 14, 15]. Therefore, the radiological diagnosis of complications is equally important to determine the type and intensity of treatment required. The modality of choice is contrast-enhanced magnetic resonance imaging in view of its superior contrast resolution for soft-tissue and marrow abnormalities. The ability to depict cross-sectional anatomy and pathology with better tissue characterization is a distinct advantage of MRI over CT scan. Contrast administration is useful to identify non-viable necrotic areas. CT scan is complementary to MRI to better demonstrate bone erosion and demineralization.

The present study is one of the largest studies conducted on imaging of COVID-19-associated ROCM. However, there are certain limitations to our study. First, we did not correlate the imaging findings with the clinical outcomes. Second, a comparison of the findings from our study with those found in ROCM not related to COVID-19 illness was not done.

## Conclusion

Patients recovering from COVID-19 illness with co-morbidities are at risk for developing sinonasal mucormycosis which aggressively invades into surrounding soft tissue, orbit, and intracranial structures. With proper knowledge of the radiological appearance of mucor infection, early identification of disease is possible through combined MRI/CT protocol. The use of contrast studies is a must to identify devitalized tissue for adequate debridement. MRI signals of sinonasal mucor lesions may vary due to variation in contents and severity of infection. Therefore, it is preferable to identify radiological signs of invasion, than to look for characteristic signals.

## Data Availability

All data supporting the findings of the current study are available within the article. Also, the datasets used and/or analyzed during the current study are available from the corresponding author on reasonable request.

## Abbreviations

ROCM: Rhino-orbital-cerebral mucormycosis
COVID-19: Coronavirus disease 2019
SARS-CoV-2: Severe acute respiratory syndrome coronavirus 2
MRI: Magnetic resonance imaging
GFR: Glomerular filtration rate
FLAIR: Fluid-attenuated inverse recovery sequence
DWI: Diffusion-weighted imaging
